# CROSS-COUNTRY COMPARISON OF INTERNET USE AND DEPRESSION BY GENDER: THE ROLE OF INTERGENERATIONAL FACTORS

**DOI:** 10.1093/geroni/igz038.1203

**Published:** 2019-11-08

**Authors:** Hyunju Shim, Jennifer A Ailshire, Eileen Crimmins

**Affiliations:** 1 University of Southern California, Los Angeles, United States; 2 University of Southern California, Los Angeles, California, United States; 3 Davis School of Gerontology, University of Southern California, Los Angeles, California, United States

## Abstract

Technology may offer one approach to reducing depression as it provides medium to maintain connections (Cotton et al., 2014). Yet, depression, internet use, gender roles, and expectation of intergenerational interaction all differ across countries. Using nationally representative data from the U.S (Health and Retirement Study: HRS) and South Korea (Living Profiles of Older People Survey: LPOPS), the study examines 1) association between internet use and depressive symptoms by gender in two countries; 2) and whether intergenerational factors moderated this association. In the U.S., more than half of men and women aged 65+ used the internet, while approximately 30% of women and 47% of men used the internet in Korea. Using the internet was associated with lower depression for those living far from the closest child for women in the U.S., and for men in Korea. The findings indicate that the association of internet use on depressive symptoms can be influenced by intergenerational factors that may differentially affect men and women depending on the sociohistorical contexts.

